# Case Report: Dual checkpoint inhibition with bevacizumab yields dramatic response in synchronous double primary HCC and ICC with lung metastasis

**DOI:** 10.3389/fonc.2025.1670818

**Published:** 2025-10-24

**Authors:** Yu-cheng He, Xin-ye Dai, Ying-hao Lv, Xiao-juan Yang, Qing-yun Xie, Si-nan Xie, Yun-shi Cai, Feng-wei Gao, Tian Lan

**Affiliations:** ^1^ Liver Transplant Center, Transplant Center, West China Hospital, Sichuan University, Chengdu, China; ^2^ Liver Digital Transformation Research Laboratory, State Key Laboratory of Biotherapy and Cancer Center, West China Hospital, Sichuan University and Collaborative Innovation Center of Biotherapy, Chengdu, Sichuan, China; ^3^ Department of Biotherapy, Cancer Center and State Laboratory of Biotherapy, and Frontiers Science Center for Disease-related Molecular Network, West China Hospital, Sichuan University, Chengdu, China

**Keywords:** synchronous double primary hepatocellular carcinoma and intrahepatic cholangiocarcinoma (sdpHCC-ICC), combination immunotherapy, PD-1, CTLA-4, bevacizumab, conversion therapy

## Abstract

**Background:**

Due to its rarity, synchronous double primary hepatocellular carcinoma and intrahepatic cholangiocarcinoma (sdpHCC-ICC) presents significant challenges for preoperative diagnosis and is lacking established systemic therapies. Here, we present an extremely rare case of unresectable collision-type sdpHCC-ICC with pulmonary metastases, who achieved significant responses in both pulmonary metastases and hepatic lesions following combination immunotherapy.

**Case report:**

This case report describes a 69-year-old male diagnosed with collision-type sdpHCC-ICC accompanied by pulmonary metastases. The patient underwent combination immunotherapy with dual immune therapy of nivolumab plus ipilimumab and bevacizumab, achieving complete remission (CR) of pulmonary lesions and partial response (PR) in hepatic lesions. Subsequent surgical resection of the residual liver tumor and postoperative adjuvant therapy resulted in favorable long-term outcomes.

**Conclusion:**

Our study reports the first case that systematic therapy achieved successful conversion therapy in an unresectable sdpHCC-ICC. Combination immunotherapy might represent a promising therapeutic strategy for sdpHCC-ICC, warranting further validation.

## 1 Introduction

Primary liver cancer (PLC) is the third leading cause of cancer death worldwide and is mainly composed of hepatocellular carcinoma (HCC) (75%–85%) and intrahepatic cholangiocarcinoma (ICC) (10%–15%) (1, 2). However, liver cancers containing both HCC and ICC components are relatively rare, account for only 2%-5% of PLC (3). In 1949, Allen and Lisa first divided this type of tumor into three subtypes (4): subtype A refers to HCC and ICC growing independently in different liver sites as separate tumors; subtype B refers to tumors formed by the two components in a continuous manner; and subtype C refers to tumors where the two components are mixed within the same lesion. In 1985, Goodman et al. proposed an alternative classification (5): type I refers to collision tumors; type II refers to transitional tumors; and type III refers to fibrolamellar tumors. According to the present consensus, Allen’s subtype C and Goodman’s type II are termed combined hepatocellular-cholangiocarcinoma (cHCC-CCA), which accounts for approximately 1.0% to 4.7% of PLC (3, 6). In contrast, Allen’s subtype A and Goodman’s type I, which have been distinguished from cHCC-CCA, are regarded as synchronous double primary hepatocellular carcinoma and intrahepatic cholangiocarcinoma (sdpHCC-ICC) and are even rarer, constituting only 0.23% to 0.8% of PLC (7–9).

However, due to its rarity, sdpHCC-ICC presents significant challenges for preoperative diagnosis and no specific clinical guidelines exist for sdpHCC-ICC treatment. Treatment decisions for sdpHCC-ICC are usually based on studies of HCC or ICC. Therefore, there’s an urgent need to develop more effective treatment strategies that target the unique features of sdpHCC-ICC. With the discovery of immune checkpoints and the application of inhibitors (ICIs), particularly programmed death-1 and its ligand (PD-1/PD-L1), immunotherapy has redefined the therapeutic landscape in cancer (10, 11). Concomitantly, emerging research has attempted to dissect the molecular mechanisms, refine multimodal regulatory strategies, and delineate the clinical applications of cancer immunotherapy (12–16). However, the role of immunotherapy in sdpHCC-ICC remains entirely unexplored.

Here, we present an extremely rare case of unresectable collision-type sdpHCC-ICC with pulmonary metastases. Combination therapy with dual immune therapy of nivolumab plus ipilimumab and bevacizumab has achieved successful conversion therapy in the patient for surgical resection.

## 2 Case presentation

**Figure 1:**
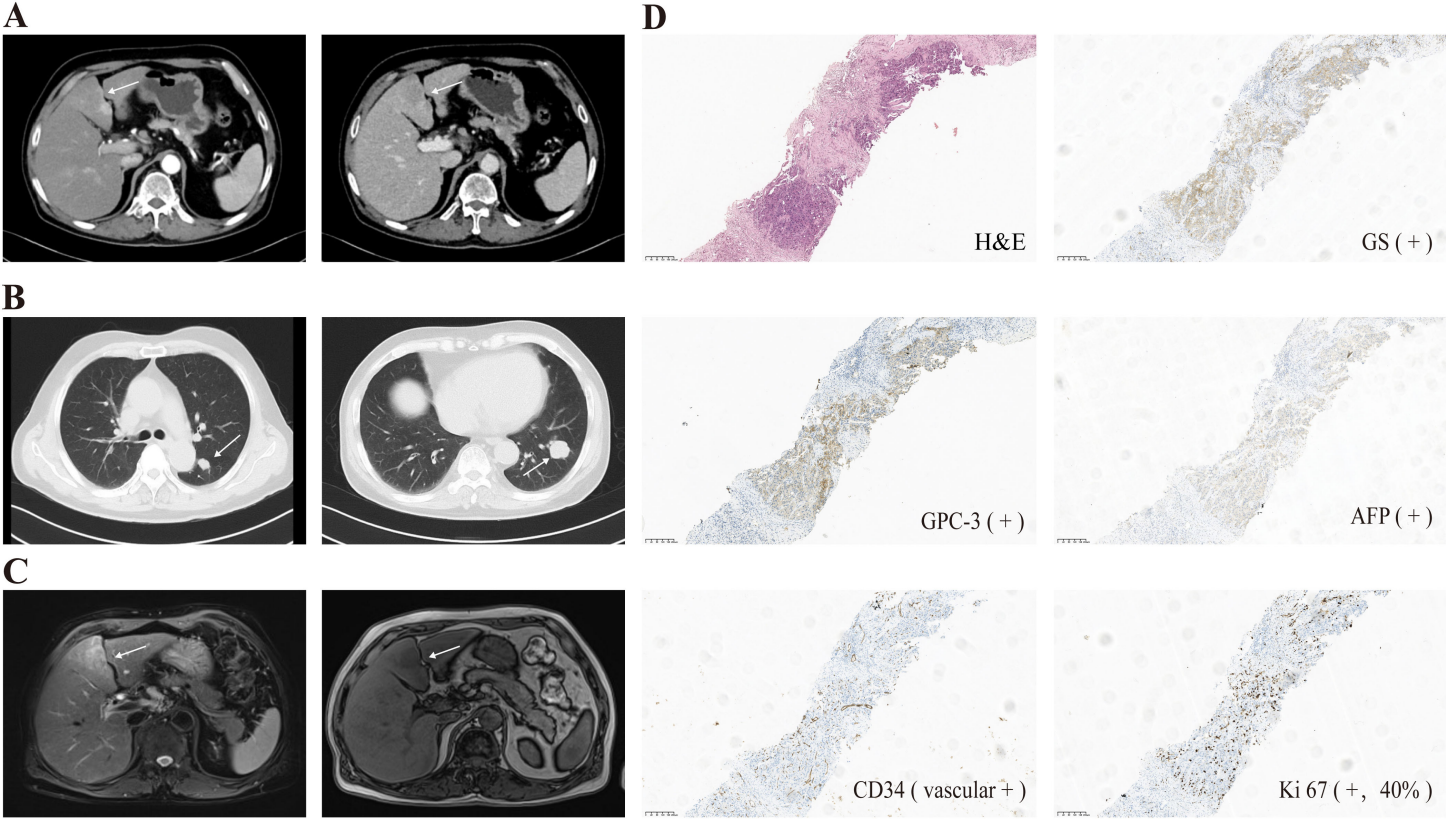
**(A)** Contrast-enhanced abdominal CT scan identified the hepatic lesions (white arrows); **(B)** Contrast-enhanced chest CT identified the pulmonary lesions (white arrows); **(C)** Abdominal MRI confirmed the hepatic lesions (white arrows); **(D)** Morphological and immunohistochemical features of the percutaneous liver biopsy (magnification, ×100).

In May 2021, a 69-year-old man was incidentally diagnosed with hepatic and pulmonary occupying lesions during an imaging evaluation for lumbar disc herniation. Contrast-enhanced abdominal computed tomography (CT) imaging (**Figure 1A**) revealed mass-like lesions within the left medial lobe and superior segment of the right anterior lobe of the liver, with the largest lesion measuring 1.4 × 1.4 cm. Simultaneously, contrast-enhanced chest CT imaging (**Figure 1B**) identified multiple lesions in both lungs, ranging from 0.4 to 2.5 cm in size, with the largest lesion located in the lateral basal segment of the left lower lobe. Laboratory investigations demonstrated that the patient’s liver function tests and tumor markers were within normal limits. The patient was also found to have compensated hepatitis B cirrhosis, with a 40-year history of chronic hepatitis B infection but no prior antiviral therapy. Additionally, he had a 30-year history of excessive alcohol consumption and smoking.

Further diagnostic examination, including abdominal magnetic resonance imaging (MRI) (**Figure 1C**) and percutaneous liver biopsy, confirmed the diagnosis of hepatocellular carcinoma (HCC) with pulmonary metastases (BCLC stage C). Histopathological and immunohistochemical analyses of the liver biopsy specimen (**Figure 1D**) revealed the following results: GS—positive, GPC-3—positive, AFP—positive, CK8/18—positive, CK7—positive, HCC —negative, CK5/6—negative, P40—negative, S-100—negative, TTF-1—negative, CD34—positive(vascular), Ki67 at 40%.

**Figure 2:**
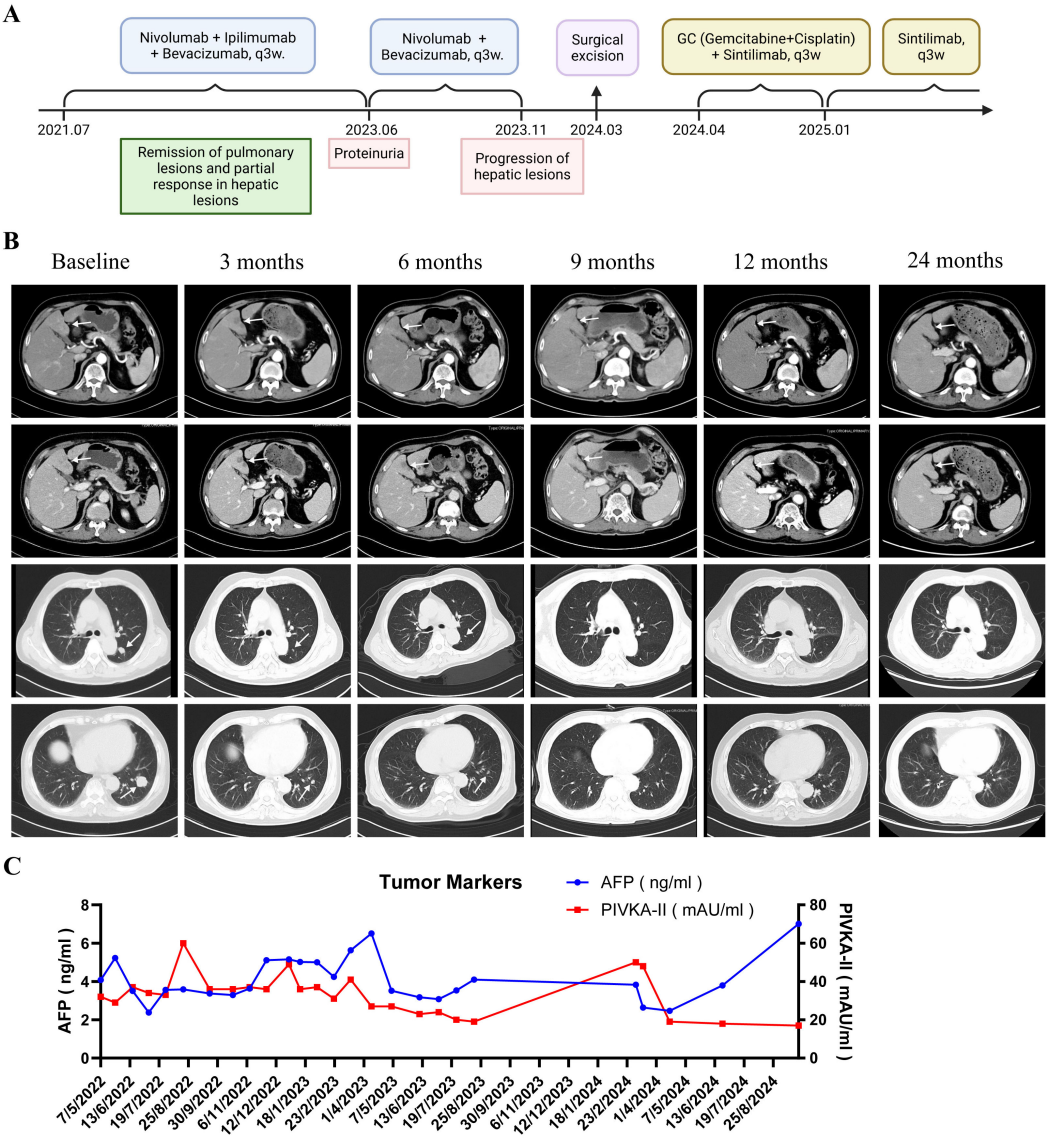
**(A)** Timeline of the whole treatment process for the patient; **(B)** Contrast-enhanced CT scans shown complete remission of pulmonary metastases and partial response of hepatic lesions (white arrows); **(C)** Laboratory examination showed normal-level AFP and occasionally mildly increased PIVKA-II. AFP, alpha-fetoprotein; PIVKA-II, protein induced by vitamin K absence or antagonist-II.

The patient received systemic therapy from July 2021, comprising dual immune therapy of nivolumab (1 mg/kg, every 3 weeks) and ipilimumab (1 mg/kg, every 3 weeks), along with the anti-angiogenic agent bevacizumab (15 mg/kg, every 3 weeks) (**Figure 2A**). By January 2022 (8 cycles), pulmonary metastases achieved complete remission (CR), while hepatic lesions showed partial response (PR) (50% reduction) (**Figure 2B**). Ipilimumab was permanently discontinued in April 2023 due to grade 3 proteinuria (CTCAE v5.0), with nivolumab and bevacizumab continued until disease progression. Laboratory examination of tumor markers showed normal-level AFP and occasionally mildly increased PIVKA-II (**Figure 2C**). HBV DNA remained undetectable (<1.00E+02 IU/mL) under antiviral therapy, with transient hypothyroidism and hypertriglyceridemia managed medically.

**Figure 3:**
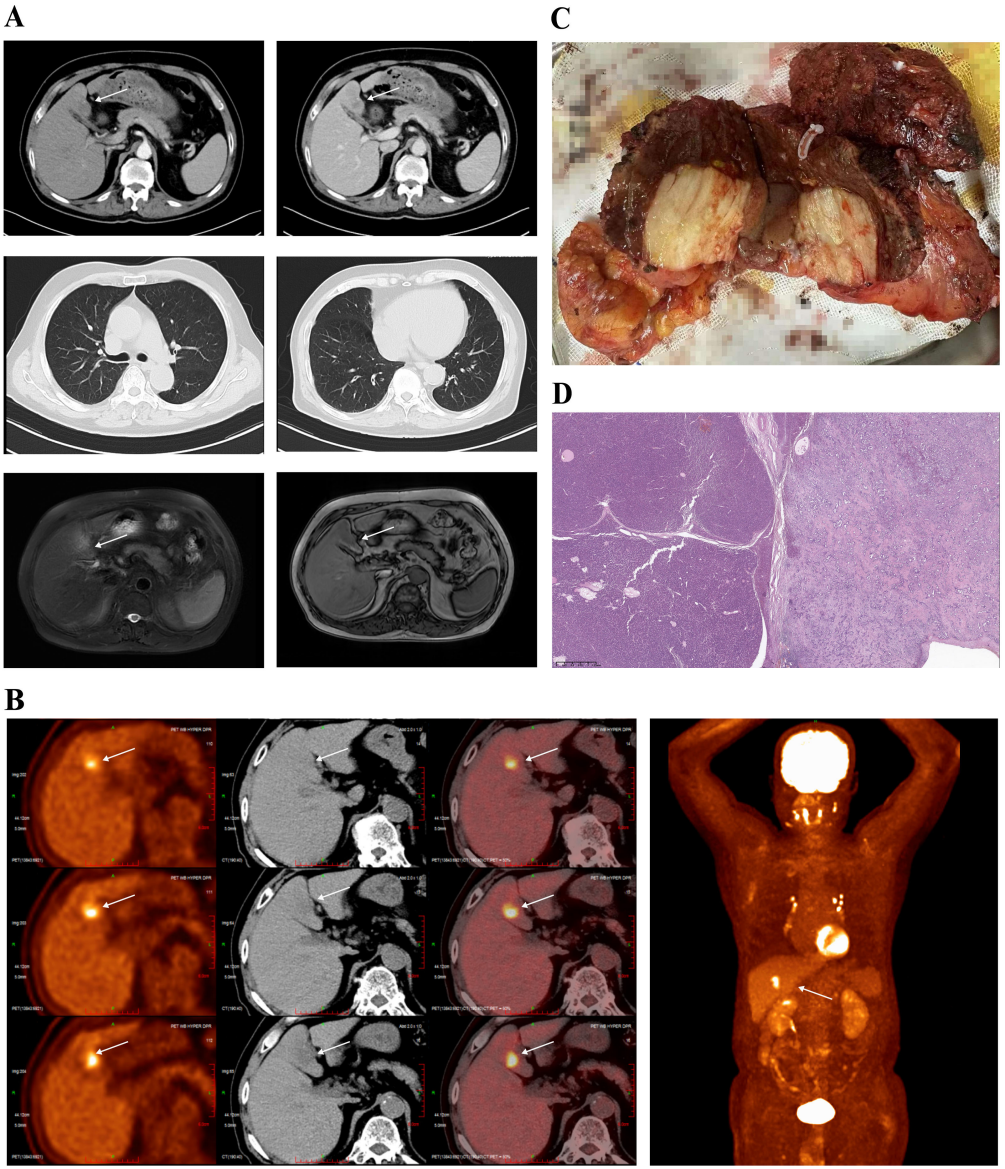
**(A)** Contrast-enhanced CT scans and abdominal MRI revealed progressive hepatic lesions despite sustained pulmonary remission (white arrows); **(B)** PET-CT confirmed the progressive hepatic lesions (white arrows); **(C)** Intraoperative view of the resected liver segment shown a lesion with two adjacent tumors; **(D)** Postoperative histopathological analysis revealed a collision-type sdpHCC-ICC (magnification, ×20).

In February 2024, six months after cessation of ipilimumab, routine imaging (**Figure 3A**) revealed progression of the hepatic lesions, a finding subsequently confirmed by PET–CT (**Figure 3B**). A multidisciplinary tumor board therefore recommended surgical resection, and on 19 March 2024 the patient underwent a laparoscopic middle hepatectomy with cholecystectomy.

**Figure 4:**
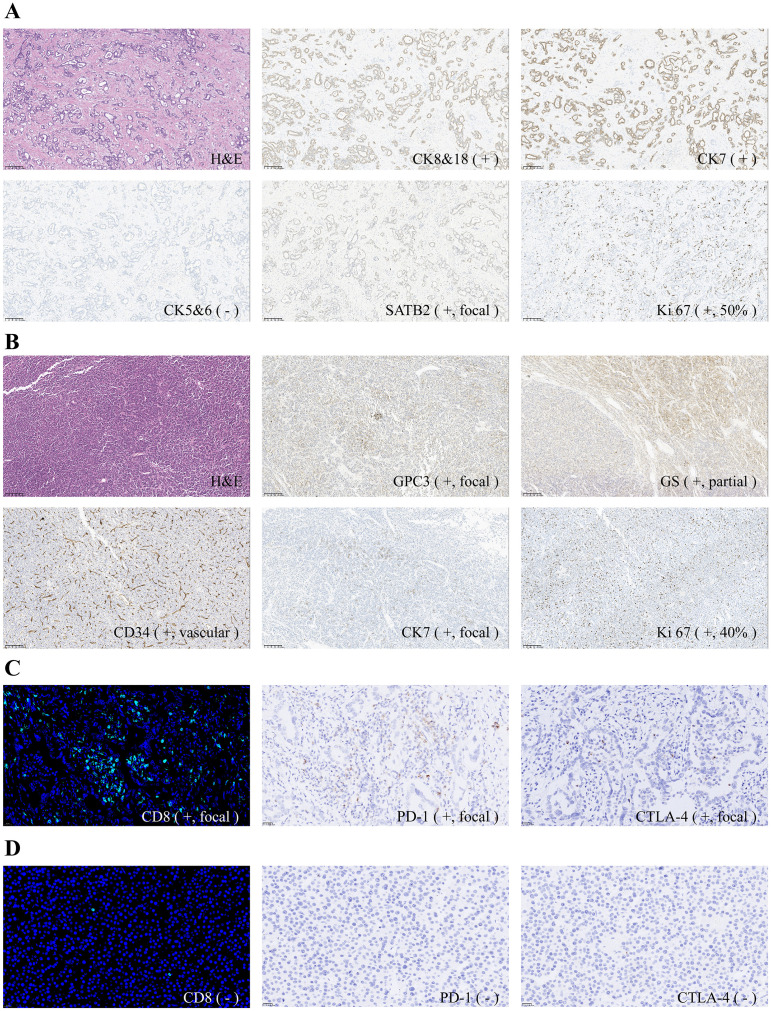
**(A)** Morphological and immunohistochemical features of tumor A (magnification, ×100); **(B)** Morphological and immunohistochemical features of tumor B (magnification, ×100). **(C)** CD8 immunofluorescence staining and PD-1/CTLA-4 immunohistochemical staining of tumor A (magnification, ×magni **(D)** CD8 immunofluorescence staining and PD-1/CTLA-4 immunohistochemical staining of tumor B (magnification, ×magni Nuclei were stained with DAPI (blue) in the immunofluorescence images.

The intraoperative view of the resected liver segment shown a lesion with two adjacent tumors (A and B) (**Figure 3C**) and postoperative histopathological analysis supports a diagnosis of collision-type sdpHCC-ICC (**Figure 3D**):

Tumor A indicated moderately differentiated (Grade 2) cholangiocarcinoma with invasion into the perihilar fat and connective tissue and microscopic neural invasion. Immunohistochemical tests (**Figure 4A**): CK8/18—positive, CK7—positive, CK5/6—negative, CK20—negative, CDX2—negative, SATB2—positive(focal), NKX3.1—negative, CgA—negative, Syn—negative, WT1—negative, Ki-67 at 50%.

Tumor B indicated moderately differentiated (Grade 2) HCC without capsular involvement. Immunohistochemical tests (**Figure 4B**): CPC3—positive(focal), GS—positive(partial), CD34—positive(vascular), Arg-1—positive, HSP70—positive, CK7—positive(focal), HepPar-1—positive, Beta-Catenin—positive(membranous), Ki-67 at 40%.

Further pathological analysis demonstrated that tumor A (**Figure 4C**) exhibited more pronounced CD8^+^ cell infiltration and higher expression levels of PD-1 and CTLA-4 compared with tumor B (**Figure 4D**).

Following surgery, the patient received adjuvant chemotherapy with gemcitabine- cisplatin (GC) for 4 cycles. Meanwhile, sintilimab (3mg/kg, q3w) was initiated one month after surgery, and regular follow-up for the condition was commenced. At one year follow-up (June 2025), imaging examination demonstrated no recurrence (**Figure 3**), and the patient maintained excellent functional status (ECOG 0).

## 3 Discussion

sdpHCC-ICC is a rare and unique primary liver malignancy with an unclear pathogenesis. Due its rarity, most cases of sdpHCC-ICC were diagnosed initially as HCCs, with only about 20% being accurately diagnosed (17, 18). The case of our report was also misdiagnosed as HCC with pulmonary metastases prior to postoperative pathological confirmation.

The preoperative diagnosis of PLC relies mainly on imaging examinations and tumor markers. HCC typically manifests as “rapid wash-in and rapid wash-out” on enhanced CT or MRI imaging, and is often associated with elevated α-fetoprotein (AFP) levels (19, 20). In contrast, ICC is characterized by “delayed enhancement” on enhanced imaging and frequently elevated carbohydrate antigen 19-9 (CA19-9) levels (21, 22). For sdpHCC-ICC, its imaging features usually combine elements of both HCC and ICC. Moreover, studies has shown that simultaneous elevation of AFP and CA19-9 (29%) is more common in sdpHCC-ICC than in pure HCC (9%) or ICC (6%) (9). However, in our study, the collision-type sdpHCC-ICC presented as a single haptic lesion on preoperative imaging, with neither of AFP and CA19–9 elevated. Moreover, percutaneous liver biopsy identified only HCC cells due to the mixed histology of the tumor, leading to misclassification as HCC. These results underscored the limitations of conventional diagnostic methods for sdpHCC-ICC, and further exploration is needed to facilitate accurate preoperative diagnosis and appropriate neoadjuvant therapy.

Surgical resection remains the preferred treatment for sdpHCC-ICC. However, due to the insidious onset of the disease, some patients are diagnosed at an advanced stage, resulting in a loss of surgical opportunities and a poor prognosis. Unfortunately, no standardized systematic therapy has been established for patients with sdpHCC-ICC, further impeding its treatment. In recent years, combination immunotherapy has emerged as a promising approach to expand the responsive population, overcome resistance, and increase efficacy of immune checkpoint inhibitors (ICIs) (12, 23). For instance, anti PD-1/PD-L1 mAbs plus anti-angiogenic agents, such as bevacizumab, has been associated with improved clinical outcomes in HCC patients (24–26), and combination chemotherapy with ICIs has shown potential as first-line treatment for advanced ICC (27). Notably, dual immune combination therapy has also yielded significant clinical benefits in these cancers. A phase 3 clinical trial demonstrates that nivolumab plus ipilimumab has achieved a longer overall survival (OS) and higher objective response rate (ORR) compared to lenvatinib or sorafenib in advanced HCC (28). This combination immunotherapy has also elicited responses in patients with ICC (29). Given these findings, combination immunotherapy might be a promising therapeutic strategy for sdpHCC-ICC.

Previous studies on sdpHCC-ICC have predominantly centered on resectable disease, with peri-operative management limited to chemotherapy or TACE and lacking details (9, 17, 30–32). To our knowledge, we report for the first time that systematic therapy achieved successful conversion therapy in an unresectable sdpHCC-ICC. The patient received combination immunotherapy with dual immune therapy of nivolumab plus ipilimumab and bevacizumab. After eight treatment cycles, the patient achieved CR of pulmonary metastases, and the intrahepatic lesions were reduced by 50%, allowing the patient for subsequent surgical resection. This successful conversion therapy reflects the potential additive or synergistic effects of the treatment regimen: CTLA-4 inhibition expanded the tumor-specific T cell clonotype repertoire and PD-1 blockade maintained the cytotoxic activity of effector T cells (33). Furthermore, anti-VEGF therapy with bevacizumab remodeled the vasculature and immunosuppressive microenvironment, facilitating lymphocyte infiltration (34). At the 1-year follow-up, the patient remained free of tumor recurrence and maintained a satisfactory quality of life. This favorable outcome highlights the potential value of this treatment regimen for similar unresectable sdpHCC-ICC cases.

Besides, the biological behavior and immune responses of HCC and ICC cells within the tumor are also related to the therapeutic effect. Previous studies indicate that the ICC component has a more significant impact on sdpHCC-ICC prognosis than the HCC component (7, 9). Our pathological analysis revealed much higher immune cell infiltration and PD-1/CTLA-4 expression levels in the ICC component than the HCC component, indicating distinct immunogenicity and immune responses to ICIs. This finding is consistent with previous observations that ICC often exhibits an “immune- hot” phenotype with high immune cell infiltration (35), whereas HCC is characterized by poor immune cell infiltration and a strongly immunosuppressive microenvironment (36). The CR of the pulmonary metastases (presumably originating from the ICC clone) further supports this differential response. These findings reinforce the necessity for intensified efforts to develop effective diagnostic methods and personalized treatment strategies for the management of sdpHCC-ICC, considering its distinct clinical and pathological features.

As immune-checkpoint inhibitors (ICIs) and combination regimens expand in both biological investigation and clinical application, immune-related adverse events (irAEs) have surfaced as their limitation for broader implementation (16, 37, 38). A systematic synthesis has revealed that grade ≥ 3 adverse events were reported in 55% of patients receiving combination checkpoint inhibition, and 46% receiving immunotherapy-chemotherapy combinations (39). In our study, the patient suffered grade 3 proteinuria during treatment, resulting in permanent cessation of ipilimumab. This withdrawal may have abrogated the ongoing antitumor response and permitted subsequent progression of hepatic lesions, underscoring the imperative for rigorous monitoring and timely, protocol-driven management of irAEs. Furthermore, recent research has demonstrated that combination of nivolumab and ipilimumab produced more extensive kidney injury than nivolumab monotherapy in humanized mouse models, highlighting the urgent need for mechanistic studies and prospective validation of strategies to prevent and treat ICI-mediated nephrotoxicity (40).

Thus, our study might provide references for an initial framework for systemic therapy in sdpHCC-ICC. Moreover, the tumor’s dual hepatocellular-cholangiocellular composition provides a unique biological platform for dissecting shared oncogenic pathways and therapeutic targets across hepatobiliary malignancies, warranting indepth mechanistic exploration. However, several limitations warrant acknowledge in our study. The generalizability of a single-case experience require validation in multicenter cohorts to assess reproducibility. Additionally, the follow-up duration was limited to one year, precluding conclusions about the long-term durability of the response. We will continue to follow up with the patient, and any new data will be shared as soon as they become available. Furthermore, although we assessed immune cell infiltration and PD-1/CTLA-4 expression in the study, more immunotherapy-relevant biomarkers, such as PD-L1 and tumor mutation burden, and mechanistic analyses required to clarify the distinct immunotherapy responses of ICC and HCC components. Finally, the lack of pathological analysis for the pulmonary metastasis restricts further evaluation of the treatment.

## 4 Conclusion

In summary, this extremely rare case of unresectable collision-type sdpHCC-ICC with pulmonary metastases achieved significant responses in both pulmonary metastases and hepatic lesions through combination immunotherapy with nivolumab, ipilimumab, and bevacizumab. Our study reports the first case that systematic therapy achieved successful conversion therapy in an unresectable sdpHCC-ICC, suggesting a potential therapeutic strategy for unresectable sdpHCC-ICC. Given the distinct clinical and pathological features of sdpHCC-ICC, further research and more patient data are needed to develop effective diagnostic methods and tailored treatment strategies.

## Data Availability

The original contributions presented in the study are included in the article/supplementary material. Further inquiries can be directed to the corresponding authors.
